# Characteristics of Marine Biomaterials and Their Applications in Biomedicine

**DOI:** 10.3390/md20060372

**Published:** 2022-05-31

**Authors:** Hengtong Zhang, Xixi Wu, Liang Quan, Qiang Ao

**Affiliations:** NMPA Key Laboratory for Quality Research and Control of Tissue Regenerative Biomaterial & Institute of Regulatory Science for Medical Device & National Engineering Research Center for Biomaterials, Sichuan University, Chengdu 610064, China; zhanght_1226@163.com (H.Z.); wuxixi9997@163.com (X.W.); quanliang@stu.scu.edu.cn (L.Q.)

**Keywords:** marine biomaterials, extraction methods, marine polysaccharides, collagen, biomedical applications, tissue engineering

## Abstract

Oceans have vast potential to develop high-value bioactive substances and biomaterials. In the past decades, many biomaterials have come from marine organisms, but due to the wide variety of organisms living in the oceans, the great diversity of marine-derived materials remains explored. The marine biomaterials that have been found and studied have excellent biological activity, unique chemical structure, good biocompatibility, low toxicity, and suitable degradation, and can be used as attractive tissue material engineering and regenerative medicine applications. In this review, we give an overview of the extraction and processing methods and chemical and biological characteristics of common marine polysaccharides and proteins. This review also briefly explains their important applications in anticancer, antiviral, drug delivery, tissue engineering, and other fields.

## 1. Introduction

The total area of the oceans is about 360 million square kilometers, accounting for about 71% of the earth’s surface area [1]. The ocean is rich in various biological resources, such as skin, bone, and shells from marine organisms, which are important sources of marine biological medical materials. However, the seas that people have explored are only small parts, and most areas remain to be developed at present [2]. Driven by environmental pollution and energy shortages, the ocean seems to be a suitable choice for finding renewable and environmentally friendly resources [3]. The discovery of a bioactive substance called sea cucumber, which was extracted from marine organisms in 1967, inspired in-depth research on marine biomaterials [1,4]. Until today, an increasing number of compounds are currently being isolated from marine organisms and proposed as novel products for biomedical related applications ranging from bioactive ingredients to biological scaffolds [2]. The progress of modern medicine is inseparable from the development of biomedical materials. 

In recent years, marine biological materials that have been paid attention to by human beings are mainly some biological macromolecules and their derivatives, such as chitin, alginate, and collagen. They do not carry the risk of zoonotic transmission, avoid religious constraints for mammals, and possess good biocompatibility, biodegradability, biological activity, and processing performance [5,6]. They can be used as scaffold materials, wound dressings, and drug carrier materials for tissue engineering products. Therefore, the development and utilization of marine biological materials is a major focus in the current direction of material research and development. With the development and progress of science and technology, the market prospect of the development and application of marine biomedical materials is increasingly broad, and more and more attention is paid to it. At present, the commonly used marine biomedical materials are mainly polysaccharides and proteins. Polysaccharides include chitin, alginate, and glycosaminoglycan, and proteins are mainly collagen. This review gives an overview of the extraction, processing methods, and chemical and biological characteristics of common marine polysaccharides and proteins and introduces their applications in anticancer, antiviral, drug delivery, tissue engineering, and other fields.

## 2. Biomaterials from Marine Organisms 

### 2.1. Chitin and Chitosan

Chitin is the second largest natural macromolecular polysaccharide in the world after cellulose, which is widely found in the fungi and algae of arthropods and lower plants in the ocean [7]. It has good biocompatibility, promotes tissue growth, has great extraction and utilization value, and has anti-inflammatory, hemostatic, and analgesic properties [7]. The chemical structure of chitin is similar to that of plant cellulose, which is a polymer composed of β-(1,4)-2-acetylamino-2-deoxy-d-glucose connected by β-1,4 glycosidic bond [8]. At present, the main raw materials for chitin production in the industry are derived from the stratum corneum of crustaceans such as crab and shrimp [9]. Chitin combines with proteins to form a complex protein network system, and calcium carbonate deposits on it to form a hard shell. Therefore, the main step of chitin production is the removal of protein and CaCO_3_ while removing fats and pigments together.

**Figure 1:**
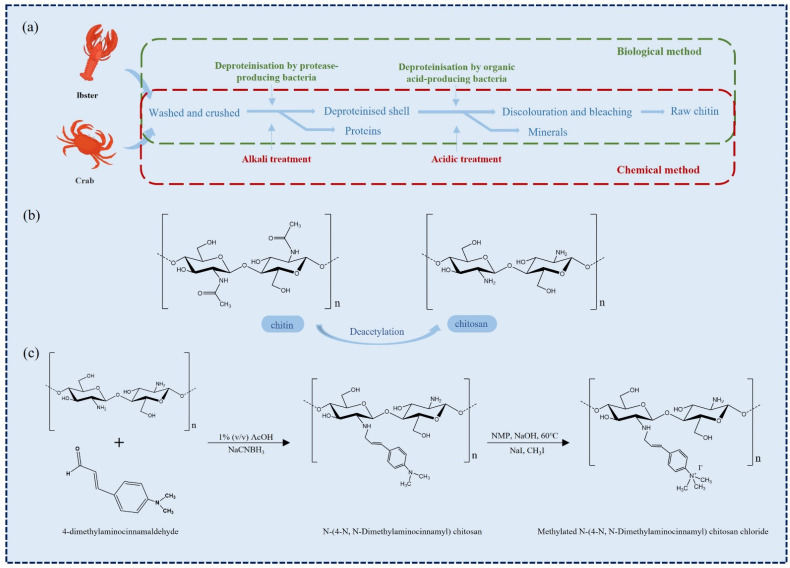
(**a**) Chitin recovery by chemical and biological methods [12]. (**b**) Structural formula of chitin and chitosan. (**c**) An instance of chitosan modification [18].

The traditional production of chitin is divided into three basic steps (Figure 1a). The first step is to remove proteins because the interaction between chitin and proteins is strong, so the deproteinization process is also the most difficult step [10]. More importantly, the degree of protein removal should be paid special attention to because excessive deproteinization will cause deacetylation and hydrolysis of chitin. On the contrary, due to the allergy symptoms of some people, protein retention will affect the application of chitin in medicine. The commonly used deproteinization reagents include NaOH, Na_2_CO_3_, NaHCO_3_, KOH, K_2_CO_3_, Ca(OH)_2_, Na_2_SO_3_, NaHSO_3_, Na_3_PO_4,_ and Na_2_S [11]. The second step is to remove inorganic salt, mainly CaCO3. The main method of desalination is acid treatment, and the desalination reagents are HCl, HNO_3_, H_2_SO_4_, CH_3_COOH, HCOOH, etc. [11]. Since CaCO_3_ is easy to be acidified, the desalination process is relatively easy to complete. To improve the desalination efficiency, it is usually achieved by increasing the temperature and the acid concentration and prolonging the desalination time, which is conducive to the immersion of acid into the chitin matrix. However, it will also bring deacetylation and hydrolysis of chitin, affecting the final quality of chitin [12]. The third step is decoloration. This is an additional step during the extraction process when the colorless product is expected, as it aims to eliminate astaxanthin and β-carotene pigments when they are present in the extraction source [13]. At present, the decolorization of chitin in the industry is mainly through sunlight irradiation, KMnO_4_ oxidation [14], and organic solvents such as acetone [15]. The sunlight irradiation time is too long, and the product is yellow (high lightness value or less yellowness index is preferable). After the decolorization of KMnO_4_, reducing agents such as NaHSO_3_ need to be added for reduction. In addition, unreacted KMnO_4_ will cause contamination of the environment. Hydrogen peroxide (H_2_O_2_) is a good choice because of its low price and pollution-free decomposition products [16].

The traditional acid-alkali treatment method also has many defects. The first is the use of a large number of acid and alkali, resulting in serious environmental pollution; meanwhile, the subsequent purification process of chitin is complex, which increases the production cost; the decrease in molecular weight and acetylation degree of chitin affects the quality of chitin products [17].

Using milder and more environmentally friendly methods to extract chitin has attracted widespread attention to overcome the environmental problems associated with acid-alkali treatment. Now a good alternative to chemical extraction of chitin is the use of various proteolytic enzymes and relatively mild ethylenediaminetetraacetic acid (EDTA), citric acid treatment. It has been found that many proteases can be used for the extraction of chitin, such as pepsin, papain [19], trypsin [20] and alkaline protease [21], etc. The mechanism of action is that the protein in the crustacean shell is separated from the crustacean shell under the catalysis of protease, and the deacetylation and depolymerization in the separation process can be minimized [12,17]. The method has the advantages of mild conditions, environmental friendliness, and high product purity [22]. 

However, compared with the acid-base method, the enzymatic method has the disadvantages of low efficiency, insufficient deproteinization of a large number of residual proteins, and high cost. Therefore, the method for enzymatic extraction of chitin needs to be improved. Weak alkali treatment under mild conditions can improve the purity of chitin and maintain its structure. The combination of enzyme extraction and mild chemical processes can improve the performance of chitin preparation. Marzieh M. N. et al. deproteinized white shrimp shell waste using trypsin and fig protease, and the enzymatic deproteinized shells were subjected to mild-alkali treatment (0–2% NaOH; 60 °C and 30 min) [19]. The results showed that the degree of acetylation (DA) of enzymatic chitin (81–83%) was higher than that of the chemical method, and the protein content was low, which could be considered a good final product. Therefore, the enzymatic method can replace the chemical method to complete the chitin extraction, which has great potential as a new protein removal method. The technology of chitin extraction by microbial fermentation has been reported [23,24]. The organic acids produced by microorganisms in the growth process were used to dissolve the minerals in raw materials such as shrimp and crab to play the role of desalination, and the produced ‘enzyme’ hydrolyzed the protein to remove it.

Chitin can be transformed into chitosan (when DA is less than 50%) under deacetylation to improve its application potential (Figure 1b). Chitosan (CHS) is one of the most widely used biomedical materials, and its structure and properties are similar to human extracellular matrix glucosamine. Under the action of lysozyme, chitosan is mainly degraded into chitosan oligosaccharide and glucosamine that can be absorbed by the human body. In addition to active hydroxyl groups, free amino groups and hydroxyl groups also exist in chitosan molecules, which makes its chemical modification more diversified, forming various chitosan derivatives and changing their properties (Figure 1c) [11]. 

To improve the yield of CHS, a mechanochemical method of direct extraction from shrimp shells is proposed [25,26]. The method applies mechanical energy (including impact, compression, shearing, friction, and stretching) to solid raw materials. With the increase in time, the solid morphology and crystal structure of substances change and induce physical and chemical reactions. Compared with the traditional acid-base method, the mechanochemical method has higher conversion efficiency and reduces the adverse impact on the environment. Moreover, the mechanical force can reduce the molecular weight of CHS and realize higher solubility. Chen et al. produced low molecular weight CHS (purity of ca. 90%) from chitin and shrimp shell by a solvent-free mechanochemical method [27]. The products exhibited a much narrower molecular weight distribution than the traditional method, with adjustable DA (40% to 83%) and MW (1 to 13 kDa) values, and base usage was reduced to about 1/10 in the extraction process [27]. A mechanochemistry method can create the possibility of large-scale production of chitosan.

As CHS cannot be dissolved in a neutral aqueous solution, it is frequently necessary to add organic or inorganic acids to dissolve CHS in the preparation of CHS-based materials. However, the β-1,4-glycosidic bond of CHS in acidic solution is prone to degradation, resulting in a decrease in molecular weight and the weakening of its antibacterial performance [28]. The poor stability of CHS-based systems limits its application in biomedicine and other fields, so how to improve the storage stability is of great significance. Several strategies to improve chitosan stability (addition of the stabilizing agent during the preparation process [29], blending with hydrophilic polymer [30], and use of ionic or chemical cross-linkers [31]) have also been reported. However, only a limited number of studies and review articles have been devoted to long-term stability studies on CHS [32].

### 2.2. Alginates

**Figure 2:**
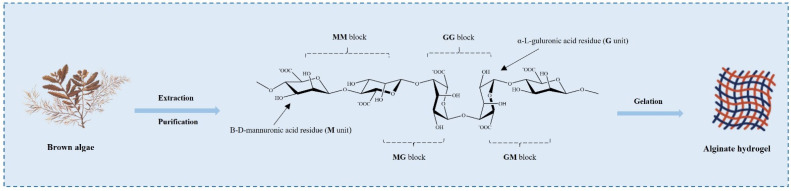
Stylized conformation structures of alginate units, blocks, and their linkages M unit: β-d-mannuronic acid residue; G unit: α-l-guluronic acid residues. Alginates are extracted and purified from various brown algae and have the gelation ability.

Alginates are natural hydrophilic polysaccharides derived from *kelp* or *Sargassum* algae of brown algae and several bacterial strains [33,34]. Chemically, alginates are mainly composed of β-d-mannuronic acid (M unit) and α-l-glucuronic acid (G unit), and different proportions of GM, MM, and GG fragments are formed via the β-1,4-glycosidic bond (Figure 2) [35]. Alginates have excellent biocompatibility, low toxicity, and the most important property is their gelling ability. When divalent cations are added to alginate in an aqueous solution, the metal ions of G units are replaced with these divalent cations, and the G units are stacked to form cross-linked networks to form hydrogels, which is the most common method for the formation of alginate gels [36]. In addition, there are several methods for preparing alginate hydrogels, including ion-interaction, covalent cross-linking, thermal gelation, and cell cross-linking [35]. By altering the type and density of cross-linking, the physical and chemical properties of the alginate hydrogels can be tailored for various biomedical applications [37,38]. However, it is clear from various studies that the application of alginate hydrogels is limited due to their poor mechanical strength and syneresis occurring during the formation of the gel [39]. Therefore, alginate hydrogels need to be combined with other biopolymers such as chitosan, gelatin, and starch groups to improve the physical and mechanical properties. For example, chitosan has a positive charge, and it can form a rigid composite gel with negatively charged alginate through strong electrostatic interaction [40]. Starch can be used as an insoluble filler in an alginate hydrogel matrix. The addition of starch creates structural support to control the shrinkage of alginate hydrogel, maintain the gel shape during freeze drying, and improve the mechanical strength of freeze-dried composite hydrogel [41].

The traditional extraction method of alginates is usually to add an alkaline solution (NaOH, Na_2_CO_3_) to extract alginate from the cell wall of algae after pretreatment with dilute acid [42]. Then the extract is filtered, and calcium chloride is added to the filtrate to precipitate alginate. The obtained alginate is decalcified by dilute hydrochloric acid, dissolved by sodium carbonate, filtered, and precipitated by ethanol. After further purification and transformation, water-soluble sodium alginate powder with better quality is obtained [43]. 

Nowadays, many eco-friendly methods are increasingly developed to improve extraction processes, such as microwave-assisted extraction (MAE) [44], ultrasonic-assisted extraction (UAE) [45], pressurized liquid extraction (PLE), and enzyme extraction (EAE) [46]. The advantage of using the enzyme to treat raw materials is to reduce the degradation of alginate by acid. At the same time, the enzyme has specificity, which can destroy the cell wall to make alginates dissolve more, improve the yield and efficiency, and reduce the use of solvents. MAE is considered to overcome the shortcomings of traditional solvent extraction. Rapid internal heating can lead to effective cell wall rupture and release of intracellular compounds into the extraction solvent during microwave treatment [47]. UAE is a relatively green process, which can reduce the extraction time and the amount of solvent used to limit energy consumption. Studies have shown that ultrasound does not affect the extraction product degradation or structural modification over a short amount of time [45]. PLE is a novel extraction technology based on using elevated temperatures and pressures to extract compounds from samples in an oxygen and light-free environment in a short time and using less solvent. Elevated temperature makes the raw materials easier to dissolve and achieve a higher diffusion rate, while higher pressure keeps the solvent below its boiling point [46].

Alginate can also be synthesized from *Azotobacter vinelandii* and *Pseudomonas* [34]. The production of alginate by bacterial fermentation is not limited and is affected by geographical environment and climatic conditions. It can be controlled and optimized on a large scale, and the ratio of M/G can be changed by changing fermentation conditions (such as temperature, PH, and culture medium concentration), which provides more clear chemical structure and physical properties for alginate [48].

### 2.3. Carrageenan

**Figure 3:**
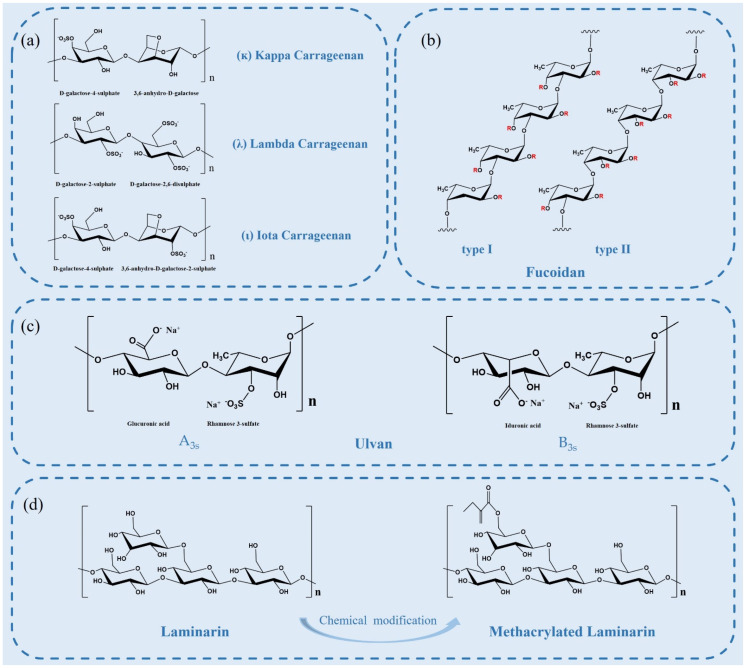
(**a**) Chemical structures of idealized repeating units of carrageenan; (**b**) two types of fucoidan backbones. R is the potential attachment of carbohydrate (α-l-fucopyranose and α-d-glucuronicacid) and non-carbohydrate (sulfate and acetyl groups) substituents; (**c**) structure of the major repeating disaccharide units that comprise ulvan; (**d**) schematic illustration of laminarin structure and its derivatives.

Carrageenan (CG) is a natural, high-molecular-weight, sulfated polysaccharide extracted from the outer cell wall and intracellular matrix of marine alga Rhodophyceae, such as Chondrus, eucheuma, Gigartina and Hypnea [49]. CGs are mainly composed of d-galactose residues linked alternately in 3-linked-β-d-galactopyranose and 4-linked-α-d-galactopyranose units. There are three major subtypes of CG: kappa, iota, and lambda carrageenan (Figure 3a), which differ in their location and number of sulfate moieties on the hexose scaffold skeleton and contain one, two, or three negatively charged sulfate ester groups per disaccharide repeating unit, respectively [50]. As the bioactive molecules, CGs have antioxidant, antibacterial, and anticoagulant properties. In addition, numerous reports have shown that CGs are also antivirals, especially anti-respiratory viruses (SARS-CoV-2, etc.) [50].

The preparation of CG usually follows such a process, including pretreatment, extraction, precipitation, filtration and drying. Firstly, the coarse impurities in red algae were removed by pretreatment, and then CG was released from cells by hot alkali extraction. Once CG is in a hot solution, it will be clarified and then converted into powder [49]. Generally, the “alcohol precipitation method” is a commonly used method to extract CG. Because of its physicochemical properties, CG is insoluble in organic reagents such as methanol and ethanol. The concentrated solution of CG is placed in alcohol to precipitate CG from the solution. Then, the solvent is evaporated, and the precipitated CG is dried and ground to the desired size [49].

Other more complex methods include extraction using enzymes [51] or fungal [52], and these biological methods can reduce the toxicity of carrageenan. In particular, some advanced extraction methods have been reported in recent years, such as deep eutectic solvents (DES) [53], MAE [54], UAE [55], and subcritical water extraction [56]. Compared with traditional methods, each extraction method may offer certain advantages, such as the improvement of the physical and chemical, gelation and biological activity conditions of CGs [57].

### 2.4. Fucoidan

Fucoidan is a complex sulfated polysaccharide, mainly derived from marine brown algae and invertebrates, such as trepang and sea urchin. Fucoidan is composed of l-fucose and sulfate groups, the main monosaccharide component of which is l-fucose-4-sulfate [58]. The structure of fucoidan is highly dependent on the algae species, but it always contains a backbone of sulfated fucans. There are two types of fucoidan backbones: type I or type II backbones (Figure 3b). One with a backbone of (1→3)-linked α-l-fucopyranose residues and the other with α-l-fucopyranose linked alternately by (1→3) and (1→4) as the main backbone [59]. Single and double substitutions in the sulfate groups at the C-2 or C-4 positions of both skeletons can occur. Some fucoidans possess substituted branches at the C-2 and C-3 positions [58]. The molecular structure of fucoidan is important to its biological activity. 

The extraction method of fucoidan will affect the structural diversity as it introduces a certain amount of bias, which results in changes in structure, charge, molecular weight and chemical composition [60]. Many classical methods can be used to extract fucoidan, such as hot water, dilute acid, dilute alkali, enzyme-assisted [61], and microwave-assisted treatment [62]. However, these methods require high temperature, a large amount of solvent or a long extraction time, and thus, researchers have proposed some new polysaccharide extraction methods to provide higher yield and maintain the natural fucoidan structure [63]. DES combined with subcritical water extraction improves the yield of fucoidan [64]; the high-pressure homogenization method can make the extraction of raccoon dog fucoid have an excellent antioxidant capacity [63]; UAE is more efficient in the extraction of higher MW fucoidan [65].

### 2.5. Ulvan

Ulvan is an acidic polysaccharide containing sulfate. It belongs to the cell wall polysaccharide of green algae that accounts for 9~36% dry weight of green algae and is mainly composed of sulfated rhamnose, uronic acid (including glucuronic acid and iduronic acid) and xylose [66]. Ulvan has been used extensively in chemical, pharmaceutical, and comestible fields because of its anticoagulation, antioxidation, antitumor, and blood lipid level reduction activities, as well as the capability of immunoregulation [1]. 

Ulvan generally has complex branching structures and no clear main chain or simple repeat unit. The main chain of Ulva pertusa from different sources is mainly composed of α-(1→4) and β-(1→4) glycosidic bonds. Glucuronic acid, edoglucuronic acid and rhamnose mainly exist in the form of aldiuronic acid in Ulvan, forming a typical repetitive disaccharide unit. The disaccharide unit mainly has two different types (Figure 3c), which is, respectively, type A ulvanobiuronic acid 3-sulfate (A_3s_) and type B ulvanobiuronic acid 3-sulfate (B_3s_) [67]. Among them, A_3s_ is mainly composed of β-d-glucuronic acid (1,4)-linked to α-l-rhamnose 3-sulfate, while in B_3s_, α-l-iduronic acid is (1,4)-linked to α-l-rhamnose 3-sulfate [67].

The extraction method of ulvan is similar to other seaweed polysaccharides, and a solvent method, physical assistant method or other method can be used. The choice of extraction method generally depends on the physicochemical properties of ulvan, which can interact with components of the cell wall when it comes in contact with a solvent [67]. Acid extraction causes almost complete desulfation of isolated ulvan, while purifying enzymatic methods maintain a significant SO_3_ substituent level [67]. UAE can destroy the cell wall and rapidly dissolve ulvan, but the structure of intracellular ulvan will not be destroyed. The degradation of ulvan may reduce or enhance its function during the extraction process, but the specific mechanism of ulvan biological activity and the structure–activity relationship between them are less studied, which requires further study [68].

### 2.6. Laminarin

Laminarin, a storage β-glucan consisting of (1,3)-β-d-glucan and some β-(1,6)-intrachain links, exists in the fronds of Laminaria and Saccharin species [69]. It has extensive biological activities, such as antitumor, antioxidant, anti-inflammatory, and prebiotic properties. Moreover, laminarins can be considered as ideal substrates for bioethanol production because they are composed of abundant glucose residues [70].

The traditional extraction method of laminarin is time-consuming and low-yield, so it is necessary to develop new environmental protection extraction technology to improve the extraction rate and yield. For example, UAE is more effective than solid-liquid extraction in extracting laminarins with higher molecular weight [71], and DES had the best extraction efficiencies for laminarin (87.6%) from marine kelp [72]. In addition, to improve the bioactivity of laminarin, various physical, chemical, and biological methods have been used to modify its structure. Catarina et al. prepared photo cross-linked laminarins by chemical modification with acrylate groups, which enhanced their applications in the biomedicine field [73]. However, only a few chemical strategies have been successfully employed due to batch variability and difficulties encountered during the modification and characterization approaches [74]. In the future, the relationship between its structure and various biological activities needs further attention and exploration

### 2.7. Hyaluronic Acid and Chondroitin Sulfate

**Figure 4:**
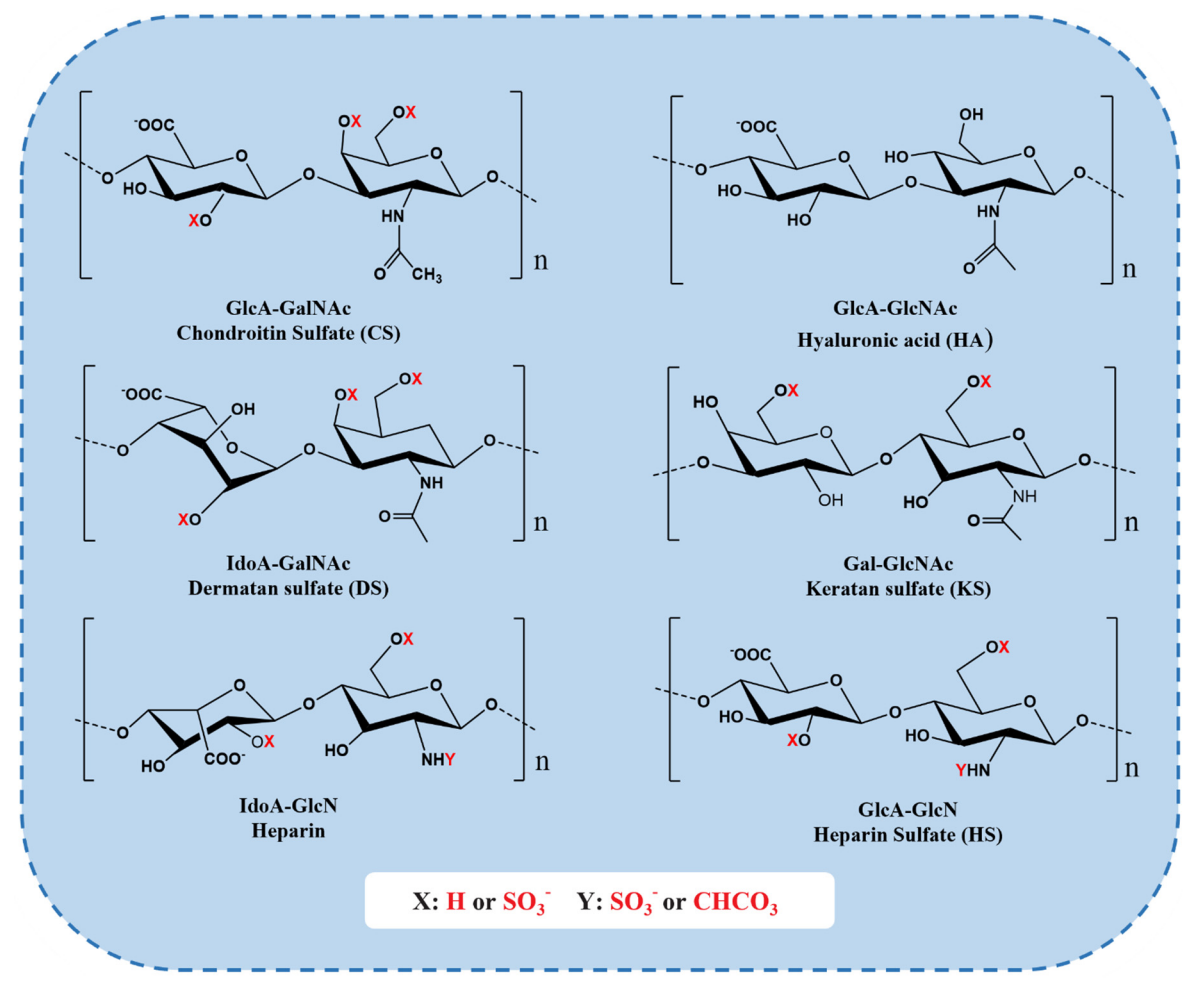
Repeating disaccharide units of different GAGs. CS chains are constituted of d-glucuronic (GlcA) and N-acetylgalactosamine (GalNAc) residues. DS is a stereoisomer of CS, including l-iduronic acid (IdoA) instead of or in addition to GlcA [76]. KS is composed of different combinations of repeating units of d-galactose (Gal) and GlcNAc [76]. HS chains comprise GlcA and d-glucosamine (GlcN). Heparin chains comprise IdoA and GlcN. These sugar residues can be esterified by sulfate at various positions as indicated by “X” or “Y” enclosed by a circle [79].

Glycosaminoglycan (GAG) is a linear polysaccharide formed by disaccharide units linked by covalent bonds and the core protein of proteoglycans [75]. The disaccharide repeat units of GAG are composed of amino sugars (including d-glucosamine and d-galactosamine) and uronic acids (glucuronic acid and l-eduronic acid) [76]. GAGs mainly exist on the surface of animal cells and extracellular matrix. Because it has a certain viscosity and lubrication function during mucus secretion, it is also called mucopolysaccharide [77]. GAGs mainly include the following categories of compounds: hyaluronic acid (HA), chondroitin sulfate (CS), heparin/heparin sulfate (HS), dermatan sulfate (DS), and keratan sulfate (KS) (Figure 4) [77]. Their differences depend on the chain length, the connection with proteins, the degree of sulfation, and the ratio of uronic acid, etc. [78]. Among them, HA and CS are two important materials that have been used in various fields such as biomedical, cosmetic, food, and drug applications.

HA is an important component of the natural extracellular matrix, which mainly exists in animal connective tissue. Unlike other GAGs, HA is unsulfated and does not bind to proteins, and it is the only GAG without proteoglycan formation and sulfate group substitution [80]. The basic units of HA are GlcA and GlcNAc, which are linked by one to three glycosidic bonds. Each disaccharide unit contains hydroxyl, which can bind to a large amount of water and interact with other extracellular proteins and polysaccharides to form a network structure. Therefore, it has the functions of water retention, lubrication, buffering, regulating osmotic pressure, and maintaining tissue morphology [81]. HS has excellent cytocompatibility and can be recognized by specific cell receptors such as CD_44_ and hyaluronic acid endocytosis receptors, thereby controlling cell adhesion, growth, and differentiation, and regulating physiological processes such as immune response, vascularization, and healing, etc. [82]. 

CS is widely present in human and animal cartilage, tendon, ligament, cornea, and vascular walls. It connects the basic unit GlcA and GalNAc by β-1,3 glycosidic bonds to form disaccharide units, which are connected by β-1,4 glycosidic bonds to form polysaccharide straight chains [83]. CS is an ideal extracellular matrix material, which can absorb and maintain water and nutrients, promote rapid cell proliferation, type II collagen, hyaluronic acid, and proteoglycan formation, and inhibit extracellular matrix degradation [84]. CS has the effects of anticoagulation, regulating blood lipids, delaying atherosclerosis, and enhancing immunity [84]. Many studies have shown that chondroitin sulfate plays an extremely important role in a variety of biological events, such as the growth of the central nervous system, damage repair, virus adhesion, growth factor signaling, morphological formation, cytoplasmic separation, and other important functions [85].

HA and CS can be extracted from different parts of marine organisms, such as cartilage, head, eyes, fins, and skin, etc. [86]. Researchers have developed and optimized technologies to decompose structures and separate GAG from other polysaccharide complexes in tissues using decontamination agents, enzymes, microorganisms, or organic solvents (mainly sodium acetate) and to ensure maximum utilization of marine wastes [87]. Due to the covalent binding between glycosaminoglycan and protein, enzymatic hydrolysis is mostly used in the extraction of glycosaminoglycan [87]. The covalent bond was broken by proteases such as pepsin, trypsin, and papain, and the glycosaminoglycan long chain was released. Helosia et al. extracted GAG from tilapia scales by papain with a yield of 0.86% [88]. GAG was purified by ion-exchange chromatography, and fraction V (FV) revealed the presence of chondroitin sulfate chains CS-A and CS-C, with a DS of 0.146 [88]. In the final stage of extraction, various purification methods (such as dialysis, ion exchange, and ultrafiltration-filtration) are generally used to separate and purify the crude glycosaminoglycan. Since different kinds of glycosaminoglycans tend to have distinct charge properties based on the extent of sulfation, Anion exchange chromatography may achieve the separation of proteoglycan species based on their glycosaminoglycan composition, at least partially [89]. For example, heparin is more highly sulfated than CS and is eluted at even higher concentrations of NaCl. Alicia M. H. et al. introduced a novel method that extracted glycosaminoglycans from articular cartilage using a combination of ethanol precipitation and enzymatic release [90]. After the separation of crude glycosaminoglycan by reverse-phase and strong anion exchange solid-phase extraction steps, a more refined disaccharide component can be obtained, which can improve its sensitivity and reproducibility in subsequent disaccharide structure analysis [90].

### 2.8. Collagen

Collagen is an important component of abundant protein and extracellular matrix in animals, which widely exists in sponges, jellyfish, sea cucumber, fish (skin, meat, scales, cartilage, fin, swim bladder, etc.), and marine mammals [91]. At present, there are more than 20 known collagens, of which type I widely exists in all tissues and organs, accounting for about 90% of the body collagen content; type II is mainly in cartilage tissue; type III exists in skin, blood vessels, and organs; type IV are in the basement membranes as a system of filtration, and type V exists in all tissues as a cytoskeleton [92,93]. These types of collagens are the most abundant and fully studied in biomedical applications [94]. 

**Figure 5:**
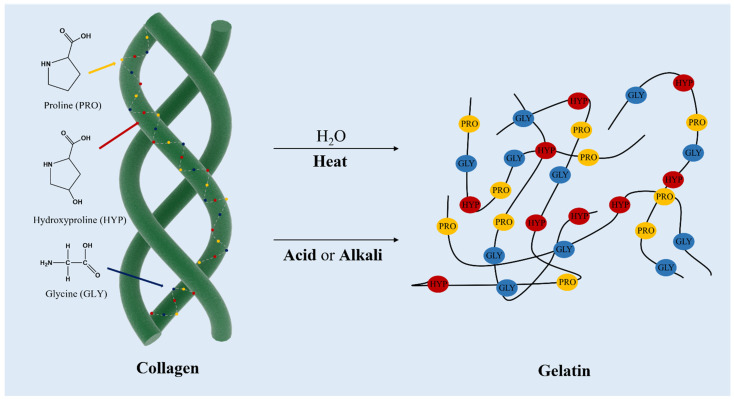
Collagen structure and the sequential amino acid contents along with the structure, and obtaining gelatin from collagen denatured by thermal and chemical treatment.

Collagen molecules are surrounded by three α-peptide chains or multi-peptide chains of α-chains to form a triple helix structure, which is arranged in a periodic fiber structure vertically and bilaterally (Figure 5) [91]. The characteristic triple helix region of collagen has high stability, so the essential amino acid composition and quaternary structure of collagen between marine collagen and terrestrial mammals are similar [95]. However, the complexity and diversity of the marine environment are higher than that of the terrestrial environment. Accordingly, the structure of marine collagen shows rich diversity with species, origin, environment, season, growth cycle and other factors, which leads to a slight difference between the composition and structure of marine collagen and terrestrial animal collagen. For many years, bovine and pigs have been used as the common source of collagens, but the outbreak of bovine spongiform encephalopathy, transmissible spongiform encephalopathy, and foot and mouth disease that happened during the last decades has limited the use of collagen from these sources [96]. The marine-derived collagen can partially replace or even replace terrestrial animal-derived collagen since both the number and the productivity of fish aquaculture species have increased in recent years. Farmed marine organisms are bred in a confined environment in which the ability to control and eventually modify (in the case of inshore facilities) physicochemical parameters could result in more homogeneous and safer collagens [97].

Based on the current research reports, the common extraction and separation technologies for different types of collagens from different sources and tissues include acid extraction [98], enzymatic extraction, hot-water extraction [99], salt extraction, alkali extraction, and fermentation (Table 1). The differences in the distribution of collagen fibers, the tightness of the binding between fibers, and the cross-linking degree between other components (such as mucopolysaccharides and minerals) in tissues and collagen fibers all affect the difficulty of collagen separation, extraction rate, purity, and integrity of collagen structure.

The acid extraction method uses low-concentration acids (commonly used hydrochloric acid, acetic acid, citric acid, and formic acid) to destroy Schiff bonds and ionic bonds so that no cross-linked collagen and collagen-containing aldehyde amine cross-linking bonds dissolve. The acid solution cannot dissolve collagen with a large cross-linking degree, and the extraction rate is not high, but it has little damage to the cross-linking bond damage of collagen, which can maintain the integrity of the collagen structure [100]. To improve the yield and reduce environmental pollution, researchers have also developed a new green extraction process. Alexandre A. Barros et al. successfully extracted sponge collagen/gelatin (about 50% extraction rate) under mild conditions by acidification of carbon dioxide into water under high pressure [101]. 

**Table 1:** Summary of collagen isolated from marine organisms.

Collagen Type	Source	Extraction Solvent or Method	Yield (Y)	Refs
Collagen type I	Tilapia Scales	A combination of dilute acetic acid (0.1 M and 0.5 M) with ultrafine bubbles	Y = 1.58%	[102]
Collagen type I	Chinese sturgeon (Acipenser sturio Linnaeus) skins	2.42% pepsin solution	The maximum yield of 86.69%	[103]
Collagen	Carp scale	300 mg/g of pepsin solution, 0.3 mol/L acetic acid solution, and 200 min ultrasonic	Y = 28.7%	[104]
Collagen type I	Scales of *Labeo rohita* and *Catla catla*	0.5 M AcOH	nearly 5%	[105]
Collagen type I and II	Skin and notochord of Bester sturgeon	M NaOH 0.1% (dry *w*/*v*) porcine pepsin	Y (type I) = 63.9 ± 0.19% Y (type II) = 35.5 ± 0.68%	[106]
Collagen/gelatin	Sponge samples of the species *Thymosea* sp.	High-pressure carbon dioxide-acidified water	nearly 50%	[101]
Collagen type I and V	Tiger puffer *Takifugu rubripes*	0.5 M AcOH and 1:20–1:50 (*w*/*w*) porcine pepsin	/	[107]
Collagen type I and V	Trash fish, leather jacket (*Odonus niger*)	0.5 mol/L AcOH and 0.1% (*w*/*v*) pepsin	Y = 64–71%	[108]
Collagen type II	Cartilages of skate and sturgeon	0.5 mol/L AcOH containing 0.1% (*w*/*v*) pepsin	/	[109]
Collagen type III	Jellyfish (*Acromitus hardenbergi*)	0.5 M AcOH (1:100 *w*/*v*), 10% (*w*/*v*) pepsin and 15 min sonication	Y (jellyfish bell) = 37.08% Y (oral arms) = 40.20%	[110]

The alkali extraction method uses alkaline substances (strong alkali or strong alkali salt) to hydrolyze the peptide bond in the material to obtain collagen. The commonly used alkalis are lime, sodium hydroxide, Ca(OH)_2_, and sodium carbonate, etc. This method is simple in operation and fast in extraction, but the relative molecular mass of the product is low. Moreover, collagen is unstable under alkaline conditions and is prone to excessive hydrolysis, which destroys the triple helix structure and even produces D-type amino acids with carcinogenic, teratogenic, and mutagenic effects [106]. Hot-water extraction is used to extract collagen by direct extraction and reflux with hot water after pretreatment of raw materials. When the water temperature is too high, the triple helix structure of collagen will be destroyed, resulting in denaturation, and the resulting product has no obvious network structure; when the temperature is too low, the extraction rate is slow and cannot even extract any collagen [111].

Enzymatic extraction is one of the relatively rational methods for extracting medical collagen. The enzymatic method has better selectivity, less damage to collagen, and mild reaction conditions required for enzymatic hydrolysis [112]. After enzymatic extraction of collagen, most of the terminal peptides have been removed, which greatly reduces its immunogenicity and is conducive to subsequent applications [113]. The principle of enzymatic extraction is to selectively remove the covalent bond between the end peptides of collagen molecules by proteases to promote the dissolution of collagen. The commonly used enzymes include pepsin, trypsin, and papain, etc. [114]. These enzymes can hydrolyze non-collagen proteins in tissues, which is convenient for subsequent salt purification and dialysis to remove these proteins. It greatly improves the purity and the extraction efficiency of collagen. In general, the factors affecting the extraction effect are not only related to the source of collagen but also related to the type of enzyme, the solid–liquid ratio, the enzyme amount, and the extraction time. In the actual extraction of collagen, to improve efficiency and yield and reduce costs, we can also combine a variety of methods, such as the acid-enzyme extraction method, which can significantly improve the yield of collagen [112,115]. This is because the diluted acid solution can effectively act on the hydrogen bond between collagen molecules so that the expansion of collagen fibers is conducive to the efficiency of enzymatic extraction. In addition, there is the enzyme-hydrothermal treatment method and the enzyme-alkali compound method [106], and these will also be combined with UAE [116], homogenization-assisted extraction (HAE) [117], extrusion [118], and other processing methods [110]. 

The extracted collagen also needs to be purified to remove impurities in collagen as much as possible, including partially modified collagen. Salting-out, dialysis, centrifugation, electrophoresis, chromatography, and ion-exchange chromatography were mainly used for purification [102,119]. Different types of collagens are separated according to the different solubility of collagen at different isoelectric points and different salt concentrations. However, it is difficult for a single method to have a good separation and purification effect, so many methods are often used in practical applications, such as the combination of ultrafiltration membrane separation technology and chromatography technology to achieve the effect of stepwise separation and purification [120].

## 3. Biomedical Applications

### 3.1. Anticancer Activity

At present, the treatment of tumor disease is mainly chemical therapy. Studies have found that most chemotherapeutic drugs can induce tumor cell apoptosis or improve body immunity, and at the same time, they also have destructive effects on normal body cells, which are harmful to a certain extent and sometimes life-threatening [121]. In recent years, with the continuous research on marine biomaterials, it has been found that many materials not only have significant antitumor activity but also have relatively small toxic side effects. Due to the special habitat, culture conditions, and separation methods of marine-derived organisms, a large number of bioactive metabolites with therapeutic activity and very unique structure can also be produced [1]. Kaori haneji et al. extracted a sulfated polysaccharide fucoidan from brown algae *Cladosiphon okamuranus Tokida* and found that it induced tumor cell apoptosis in two different leukemia cell lines [122]. Cunzhi Lin et al. found that the holothurian GAG extracted from the body wall of sea cucumber can promote the apoptosis of lung adenocarcinoma A549 cells and inhibit the proliferation of A549 cells [123]. Further studies showed that GAG could promote cell cycle arrest in G1 and G2 phases and improve the chemotherapeutic effect of cisplatin on A549 cells [123].

It is reported that chitin, chitosan, and their derivatives have the effects of adjuvant immunity, inhibition of cancer cells, and tumor growth. F.A. Taher et al. prepared chitosan from chitin extracted from raw red swamp crayfish Procambarus clarkii exoskeleton [124]. The results showed that the synthesized chitosan (higher DA) and its nanoparticles exhibited higher antitumor activity on human breast cancer SK-BR-3 and MDA-MB-231 cell lines than chitosan directly extracted from different parts [124]. The DA and particle size of chitosan are the key factors that inhibit the growth of cancer cells, and its mechanism can inhibit the proliferation of cancer cells by reducing the number of cells in the S phase and inhibiting DNA synthesis. There are also some insufficient studies on the anticancer mechanism of chitosan. Lifeng Q et al. proved that chitosan nanoparticles could inhibit the proliferation of human liver cancer BEL7402 cells, which was achieved by neutralizing its surface charge, penetrating the cell membrane, reducing mitochondrial membrane potential, and inducing lipid peroxidation in vitro [125]. Laure G. et al. showed that chitosan could directly kill cancer cells by initiating the apoptosis mechanism and improving the proliferation of T-lymphocytes [126]. By regulating some special apoptotic proteases such as Caspase-3/caspase-9, chitosan induced an up-regulation of mitochondrial pro-apoptotic protein Bax and a down-regulation of anti-apoptotic proteins, such as Bcl-2 and Bcl-XL, to reduce the adhesion between cancer cells [126]. Although these studies have shown that marine biomaterials have good therapeutic effects in mouse tumor models, this does not mean that the same therapeutic effect can be achieved in human tumors. Therefore, it is necessary to establish a model more similar to human tumors for research. In addition, whether the preparation process of biomaterials is simple and easy also needs further exploration.

Laminarin possesses a wide range of biological activities, including antitumor, antioxidant, anti-inflammatory, and other biofunctional activities [70,74]. Lin Tian et al. evaluated the antitumor roles of laminarin from seaweed (*Laminaria japonica*) [127]. The results showed that laminarin significantly inhibited the proliferation and promoted the apoptosis of BEL-7404 and HepG2 cells in vitro, and it also significantly inhibited the growth of the tumor in Hepa 1–6 tumor-bearing mice [127]. The regulatory mechanism of Laminaria in tumors is to increase the expression of senescence marker protein-30 (SMP-30) in BEL-7404 and HepG2 cells. Moreover, laminarin possesses the potential as adjuvant for cancer immunotherapy. Song et al. evaluated the effects of laminarin on the maturation of dendritic cells and on the in vivo activation of anticancer immunity [128]. The results indicated laminarin enhanced ovalbumin antigen presentation in spleen dendritic cells and promoted the proliferation of OT-I and OT-II T cells [128]. It also induced the maturation of dendritic cells in tumor-draining lymph nodes and protected interferon-γ and tumor necrosis factor-α and proliferation of OT-I and OT-II T cells in tumors [128]. Fucoidan has significant antiproliferative effects on a variety of tumor cells. Many studies have indicated that fucoidan binds to a variety of receptors on the surface of the cell membrane and causes apoptosis of tumor cells by activating the endogenous (mitochondrial and endoplasmic reticulum pathway) and exogenous (death receptor pathway) signaling pathways [129,130]. Moreover, Atashrazm et al. also found that fucoidan could enhance the ability of natural killer (NK) cells and T cells to kill tumors via activating the immune system and promoting the production of interferon gamma [131]. However, the therapeutic application of these sulfated polymers is still in the experimental stage, and their effectiveness and real efficacy remain to be observed. 

### 3.2. Antiviral Activity

In recent years, various viral diseases, such as SARS and avian influenza, have been emerging, which pose a great threat to human health. Traditional antiviral drugs often have side effects or are prone to drug resistance and other shortcomings. It is urgent to develop new antiviral drugs with small side effects and good antiviral activity. Sulfated polysaccharides extracted from marine organisms, such as carrageenan and ulvan, have received extensive attention. The antiviral activities of these bioactive substances need to be optimized by high sulfation and low molecular weight. 

**Figure 6:**
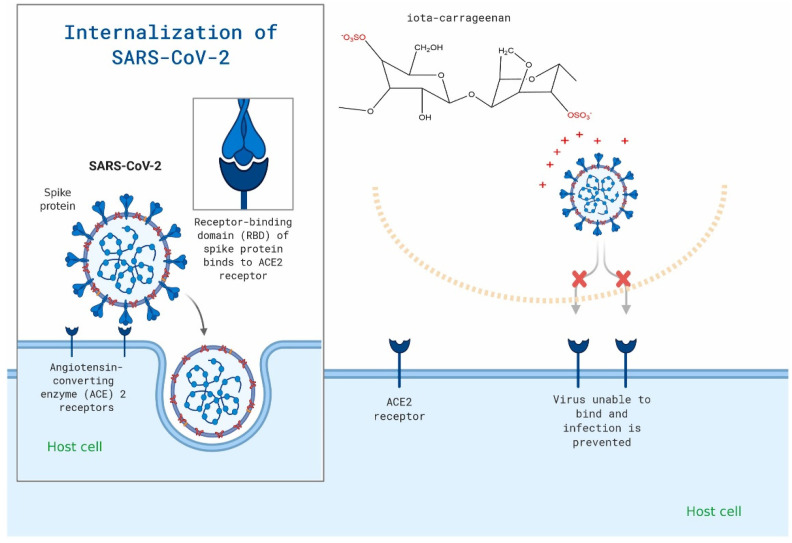
Carrageenan may be used to specifically target the viral attachment of SARS-CoV-2. The figure is reprinted from Ref. [50] with permission from the publisher.

Some studies have summarized that carrageenan and ulvan are selective inhibitors of several viruses, including herpes simplex virus (HSV), human papillomavirus (HPV), and varicella-zoster virus (VZV), etc. [66,132]. The first step of virus invasion into cells is to bind to the cell surface through electrostatic interaction and change from unstable, reversible binding to stable irreversible adsorption to realize the subsequent invasion process. These compounds could interfere electrostatically with the positively charged region of viral glycoprotein and the negatively charged HS chains of the cell receptor (Figure 6) [133]. Nayara et al. found SU1F1, a modified polysaccharide from ulvan, showed significant anti-HSV-1 activity with the highest selectivity index [133]. Romain et al. proved that carrageenan with high sulfate content and low molecular weight showed greater anti-HSV-1 activity [54].

Many authors also proposed to improve the antiviral effect of these marine bioactive substances by combining them with other drugs, including prodrug oseltamivir and zanamivir. M. Morokutti et al. found that the combination of kappa and iota-carrageenan with zanamivir in the treatment of H7N7 infected C57Bl/6 mice revealed synergistically elevated survival of mice in comparison to both single therapies [134]. Sahar et al. evaluated the antiviral selectivity of griffithsin and carrageenan against SARS-CoV-1 and SARS-CoV-2 using a cytotoxicity assay and a cell-based pseudoviral model [135]. This synergistic in vitro activity of griffithsin and carrageenan suggests that it might be useful in preventing or treating infections caused by SARS-CoV-1 or SARS-CoV-2 (half-maximal effective concentration between 3.2 and 7.5 µg/mL) [135]. In addition, many studies have shown that these sulfated polysaccharides can be used to treat and prevent coronavirus 2019 [57,136,137]. However, there is a lack of data on drug–drug and drug–disease interactions, and further studies are needed to verify the universality of these therapeutic results. The research on the antiviral mechanism of sulfated polysaccharides is still not in-depth or perfect. These studies will further promote their application in the development of antiviral drugs so as to discover low-toxic and efficient antiviral marine drugs.

### 3.3. Drug Delivery

**Figure 7:**
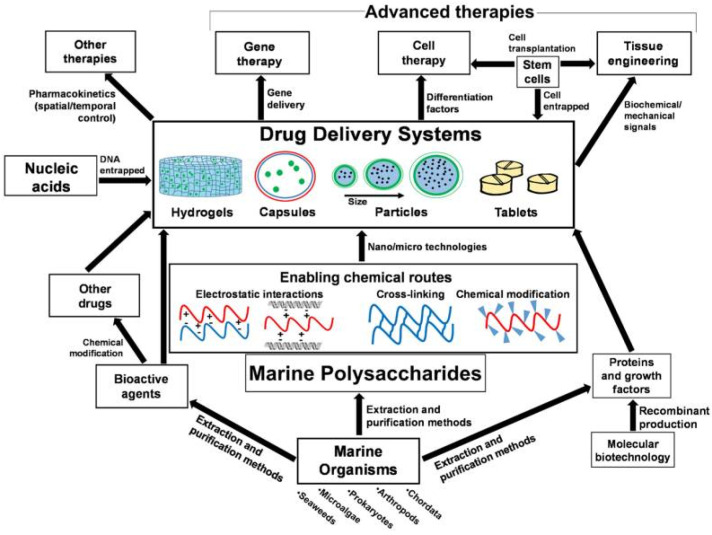
Interrelations of marine origin polysaccharides in drug delivery systems for advanced therapies and applications. The figure is reprinted from Ref. [138] with permission from the publisher.

Marine-derived materials play an important role in drug delivery applications and have been widely used as carriers for antitumor drugs, genes, and proteins [138]. At present, the bioavailability of drugs is low in the process of using drugs to treat diseases because of their inability to be effectively absorbed, their inability of effectively reaching the designated action site, and excessive metabolism in the body [139]. Moreover, multiple administrations will also increase the burden on patients, resulting in poor patient compliance. Therefore, finding appropriate drug carriers can promote drug absorption, prevent premature drug inactivation, delay drug release, and enhance drug targeting and bioavailability. Most marine polysaccharides can be modified to allow processing into various shapes and sizes, and may exhibit response dependence on external stimuli, such as pH and temperature [138]. Due to these characteristics, these biomaterials are used to construct drug delivery, including hydrogels, capsules, particles and tablets (Figure 7).

Peptide protein drugs are easily degraded by enzymes in vivo, with poor permeability and stability. Carrier encapsulation can protect proteins and promote the contact between drugs and biofilms well, thereby improving the bioavailability of drugs. Martina Nicklas et al. developed an aqueous gastro-resistant coating dispersion on the base of freeze-dried sponge collagen 15% (*w*/*w*) as the film-forming agent [140]. The results showed that tablets coated with sponge collagen resisted 0.1 M hydrochloric acid for more than 2 h and disintegrated within 10 min in phosphate buffer solution (pH 6.8) [140]. The coated tablets have good mechanical properties and can be stored for at least 6 months without loss of enteric properties. Huang YC et al. prepared pH-sensitive nanoparticles by an ionic gel method using chitosan and alginate as raw materials [141]. They used curcumin as a model drug to test the encapsulation and release and found that the encapsulation efficiency of curcumin in nanoparticles could reach 90%. In vitro studies showed that the loaded curcumin was prevented from releasing at a pH of 1.2 but was significantly released at a pH of 7.0 [141]. However, these studies lack safety assessments in complex in vivo environments, and the efficiency of the carrier delivery of drugs to specific sites needs further investigation. MAITY et al. prepared chitosan/alginate nanoparticles that exhibited significant naringenin entrapment of >90% and pH-responsive slow and sustained release of the flavonoid [142]. In vivo studies revealed a significant hypoglycemic effect after oral delivery of the nanoparticles to streptozotocin-induced diabetic rats and prevented iron-mediated oxidative stress induced by saccharification [142]. Fucoidan has been reported to target P-selectin expressed on metastatic cancer cells. Lu et al. developed a multi-stimuli-responsive nanoparticle (NPs) self-assembled via fucoidan and protamine [143]. The NPs was stable at a pH of 7.4, but enzymatic digestion and acidic intracellular microenvironment (pH 4.5–5.5) in cancer cells triggered the release of an anticancer drug (doxorubicin) from the nanoparticles [143]. The NPs with P-selectin mediated endocytosis, charge conversion, and stimuli-tunable release properties showed an improved inhibitory effect against tumor cells [143]. The pH-dependent release profile makes intravenous injection the best route for administration of these nanoparticles. To further improve the tumor-targeting function of a PH-sensitive drug carrier, targeting ligands can be modified simultaneously on the carrier to achieve tumor targeting and controllable delivery of drugs. Moreover, the stability of drug carriers, degradation behavior in vivo, and interaction with cells still need further study.

The transport of drugs into cells is one of the key steps for drug efficacy, and some characteristics of marine biomaterials can help solve these problems. For example, HA can specifically target CD_44_ receptors overexpressed in many tumor cells [144]. The chitosan ligand interacts with the receptors on the membrane surface to open the tight connection between cells so that the target drug can pass through the membrane cells [145]. Wang T. et al. designed a nano-drug delivery system in which HA-coated chitosan NPs promoted the drug delivery of 5—fluorouracil (5—Fu) into tumor cells that highly expressed CD_44_ and found that HA-chitosan NPs could effectively promote the enrichment of 5—Fu in A549 tumor cells with high expression of CD_44_ [146]. In vitro experiments showed that HA-chitosan NPs selectively bound to CD_44_ receptors and accelerated the internalization of NPs into cells [146]. The nano-drug delivery system can improve the uptake of drugs by tumor cells through targeted administration, thereby improving the antitumor efficiency of drugs. Jang B. et al. synthesized fucoidan-coated CuS nanoparticles via using the layer-by-layer technique and alternating poly-cationic and anionic substances [147]. Obtained nanoparticles not only improved the intracellular transport of fucoidan, which possesses the ability to induce apoptosis, but also provided favorable photothermal features [147]. These nanocarriers exhibit excellent drug delivery efficiency and therapeutic effect, but they lack standardized preparation methods and clinical evaluation experience. In future research, multiple responsive, intelligent carriers with less toxicity and better biocompatibility should be optimized or searched.

### 3.4. Tissue Engineering and Regenerative Medicine

**Figure 8:**
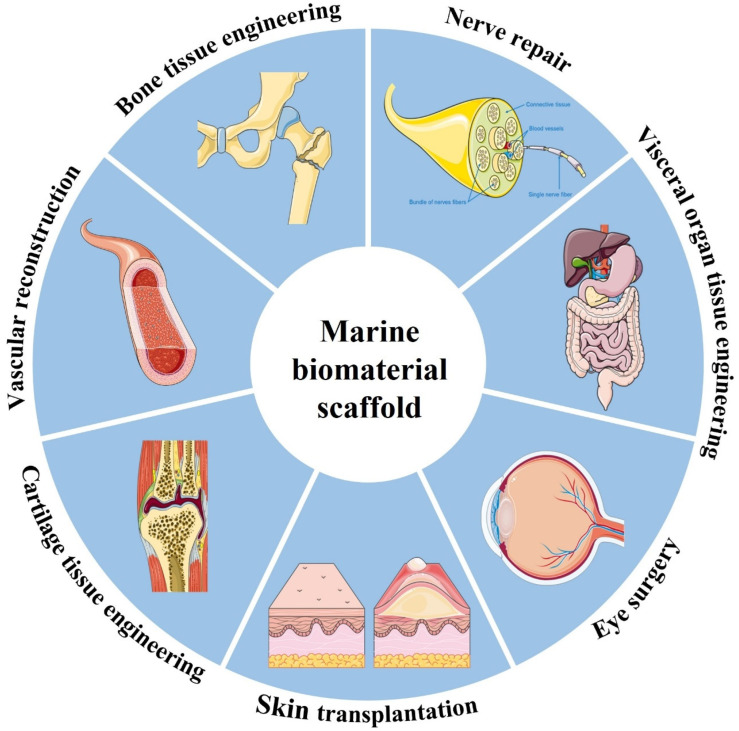
Schematic showing the wide range of applications for marine biomaterial.

Tissue engineering is one of the most important fields of biomedicine, which assembles biological materials, cells, and bioactive factors to construct transplants for tissue replacement and repair [148]. This technology helps to restore, maintain or improve the function of damaged or lost tissues or organs due to trauma or disease. Marine biomaterials have been widely used in biomedical tissue engineering and are being continuously excavated due to their low immunogenicity, good biocompatibility, promoting cell migration, inducing the interaction of the cell matrix and tissue regeneration, and forming multifunctional scaffolds with additional mechanical strength and stability through self-aggregation and cross-linking. Among them, tissue engineering scaffolds are a hot topic in current research (Figure 8). Bioscaffolds are not only the basic framework for cell adhesion but also can be used as a site for cell proliferation and differentiation. In recent years, with the emergence of some new technologies and strategies, biological scaffolds have played an important role in the construction of bionic tissues and organs. For example, 3D bioprinting can make the microstructure of scaffolds more advanced and accurate in anatomical features, which helps to more accurately co-deposit cells and biomaterials [149]. The application of marine materials in biomedical tissue engineering is mainly introduced below (Table 2). 

#### 3.4.1. Bone and Cartilage Tissue Engineering

**Figure 9:**
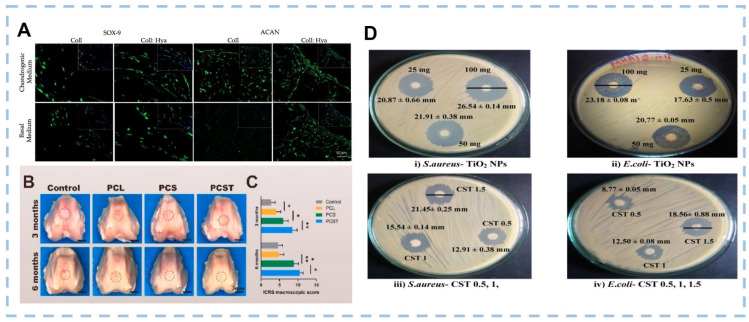
(**A**) Immunofluorescence detection of representative chondrogenic-related markers, SOX-9 and ACAN under basal and chondrogenic conditions after 21 days of culture on the Coll and Coll: Hya structures (Scale bar: 50 μm). (**B**) Representative microscopic observation of the repaired tissues at 3 and 6 months postoperatively. Red circles indicate the defect area. (**C**) ICRS score for macroscopic assessment. Data are presented as the mean ± SD (n = 8). (**D**) Antibacterial activity of TiO2 nanoparticles and scaffolds. ((**A**) was reproduced from Ref. [170] with permission from the publisher; (**B**,**C**) were reproduced from Ref. [171] with permission from the publisher; (**D**) was reproduced from Ref. [174] with permission from the publisher).

Articular cartilage is a tissue that cannot be naturally regenerated due to its special physiological structure without blood vessels and lymphatic vessels. The articular chondrocytes around the injury site undertake the task of replacing necrotic cells and reconstructing the secretory matrix. Therefore, simulating the fine structure and composition of the natural extracellular matrix is one of the important principles of cartilage tissue engineering scaffold design. Gabriela et al. fabricated highly interconnected porous 3D scaffolds made of shark skin collagen and HA [170]. In vitro studies reveal that human adipose stem cells adhere abundantly to the constructs, and the mRNA expression encoding chondrogenic-related markers such as Coll II and Sox-9 that are markedly upregulated at an early stage for both conditions, with and without exogenous stimulation (Figure 9A) [170]. This suggests the early chondrogenic differentiation of those cells. Li et al. designed a cartilage regeneration system consisting of chitosan hydrogel/3D-printed poly(ε-caprolactone) (PCL), which also contains synovial MSCs (SMSCs) and recruiting tetrahedral framework nucleic acid (TFNA) [171]. The 3D printed PCL scaffold provides basic mechanical support. Chitosan binds to DNA through electrostatic action and collects free TFNA in vivo after intra-articular injection. TFNA provides a good microenvironment for the proliferation and cartilage differentiation of transported SMSCs, promotes cartilage regeneration and greatly improves the repair of cartilage defects (Figure 9B,C) [171]. The design of the composite structure effectively avoids the structural damage of cartilage scaffold under actual load and also takes into account the diffusion ability of nutrients and bioactive factors in hydrogels. However, the adverse effect of scaffold degradation on tissue healing should also be taken into account due to the inconsistent degradation rates of PLA and chitosan. Although bone tissue has extensive regeneration ability after trauma, large bone defects cannot be recovered without intervention [172]. Various applications of marine biomaterials for bone regeneration and bone defect repair have been deeply investigated in recent years. Yash M.K. et al. designed a hybrid growth factor delivery system consisting of an electrospun nanofiber mesh tube for guiding bone regeneration with peptide-modified alginate hydrogel injected into the tube to sustain the release of growth factors [173]. By providing sustained and local release of rhBMP-2 and allowing strong cell infiltration, the alginate/nanofiber mesh system provides an environment conducive to bone regeneration and can bridge the 8 mm segmental defect in the rat femura [173]. Balu K.S. et al. produced a biomimetic nanocomposite scaffold consisting of chitosan/sodium alginate (4:4) blended with several concentrations of hydrothermally prepared titanium dioxide nanoparticles using the solvent casting approach [174]. The composite scaffolds had appropriate physicochemical and mechanical properties and showed good antibacterial properties (Figure 9D) and antiproliferative properties against the osteosarcoma MG-63 cell line [174]. Adérito JR Amaral et al. designed a dynamically cross-linked hydrogel bio-ink for 3D bioprinting [175]. The bio-ink exhibited suitable rheological properties, improved mechanical properties, and could provide appropriate microenvironments to maintain cell function. In vitro experiments showed that MC3T3-E1 cells remained viable (more than 90%) after bioprinting for at least 14 days and displayed uniform cell distribution, which indicated that the cells could withstand the mechanical stress and pressure exerted on them during the printing process [175]. 

A wide variety of marine biomaterials have been explored to produce tissue engineering scaffolds able to regenerate the bone and cartilage [176]. It is worth emphasizing that most studies only performed a short-term evaluation after material implantation [177]. Hence, the long-term biological performance of the marine biomaterial scaffolds needs to be further determined to verify that these combinations are safe and effective materials.

#### 3.4.2. Skin Tissue Engineering 

Skin transplantation is still a traditional gold-standard treatment method for large area skin injury caused by physical trauma or chemical burns, but the problems of insufficient supply, scar, pain, and infection risk make the transplantation complicated [178]. Marine biomaterials can play an important role in skin tissue repair. They have obvious biological activities and can replace the damaged skin, provide temporary barrier functions and avoid wound healing [179]. Pallabi Pal et al. extracted type I collagen from the scales of mrigal fish (Cirrhinus cirrhosus) and freeze-dried it into porous sponges [180]. The expansion rate of sponges was about 410% and the degradation rate was 18 days. In vivo experiments found that on the 15th day, even though the control wound had unhealed the de-epidermized region, the collagen-treated group had completely healed all dermal components, mature collagen matrix, and stratified epidermis [180]. However, these single component biomaterials lack effective control in mechanical properties, water absorption capacity and sensitivity of internal and external stimuli, so their application performance contains certain limitations. HuanCao et al. prepared fish collagen/chitosan/CS scaffolds using the freeze-drying method and combined them with polylactic acid-co-glycolic acid microspheres loaded with a basic fibroblast growth factor [181]. In vitro experiments showed that the scaffold had good biocompatibility and could promote fibroblast proliferation and skin tissue regeneration [181]. Current research has sufficiently proven that marine-derived biological macromolecules such as collagen, alginate, chitin, chitosan, and other molecules have significant abilities to enhance the healing process and reestablish skin tissue [178]. Sellimi et al. investigated the topical application of a cream based on the brown algae *Cystoseira barbata* laminaran [182]. It could improve the wound healing process in rats by accelerating the collagen deposition and increasing fibroblast and vascular densities, as well as protecting the cells against free radical oxidative damage [182]. Although these skin repair biomaterials have certain wound repair ability, it is still difficult for most materials to achieve the perfect regeneration of skin composition, structure, and function. In addition, the skin repair process cannot be monitored in real-time, and thus, it is difficult to obtain feedback on abnormal conditions. Therefore, improving the timeliness of wound monitoring and repair and realizing complex diagnoses as well as special treatment functions will be important trends in the development of skin repair materials in the future.

#### 3.4.3. Nerve Tissue Engineering

Peripheral nerve injury is caused by various reasons, such as sensory disorder, motor disorder, and nutritional disorder in the area dominated by the nerve. When the damaged nerve gap is too large to repair through the end-to-end connection of the damaged nerve stumps, a nerve graft is needed to bridge the gap and guide the growth of nerve fibers [183,184]. Autologous nerve transplantation is the gold-standard technique for repairing peripheral nerve injury. However, due to the limitations of tissue availability, donor site morbidity, secondary malformation, and potential differences in tissue structure and size, synthetic nerve conduits seem to be a more appropriate choice. Marine biomaterials have great application potential in nerve repair due to their better biocompatibility, cell affinity, and non-cytotoxicity. Jiang et al. prepared a nerve graft consisting of microporous chitin conduits and internal carboxymethyl-CHS fibers using the solution coating method [185]. This nerve transplantation was applied to the sciatic nerve bridge on a 10 mm defect in SD rats and could effectively promote the recovery of damaged neurons, which was similar to the effect of autologous transplantation [185]. Itai et al. prepared a CHS/collagen hydrogel catheter consisting of two coaxial hydrogel layers of chitosan and collagen through molding and mechanical anchoring [186]. The CHS layer mechanically strengthened the catheter, while the collagen layer provided a scaffold for cells supporting axon extension, and the conduit properly induced the axonal extension of the neuron cells in the conduit direction using the cellular support from the cells in the conduit [186]. To help patients achieve functional recovery as much as possible, marine biomaterials in scaffolds are mainly functionalized to improve their binding specificity, with different ligands and compounds such as growth factors and neurotrophic factors, or seeded with different cells such as mesenchymal cells or neural stem cells [187]. In conclusion, these marine biomaterials can effectively support nerve repair and regeneration, but the optimization of nerve function recovery remains to be further studied. The reported literature focused more on the nerve regeneration in nerve grafts and local effects of implanted biomaterials and lacked systematic research on overall functional recovery. Therefore, more reasonable animal models and optimal design were needed to support clinical treatment.

#### 3.4.4. Other Biomedical Applications

The wide application of marine biomaterials also includes the treatment of osteoarthritis, diabetes [188] and diabetic foot ulcers [189], anti-inflammatory materials [190], and some applications in organ tissue engineering such as liver regeneration [191], intestinal replacement [192,193], tendon regeneration [194], and myocardial regeneration [195], etc. Kyojin Kang et al. fabricated alginate hydrogel and mouse-induced hepatocyte-like cells (miHeps) dispersed in hydrogel and generated a 3D hepatic scaffold via 3D bioprinting [191]. The results showed that the hepatic scaffold expressed albumin, and ASGR1 and HNF4a expression gradually increased for 28 days in vitro [191]. The cells in hepatic scaffolds implanted in vivo grew more and exhibited higher albumin expression than in vitro scaffolds [191]. It was demonstrated that the scaffolds facilitated hepatic cell proliferation without the loss of hepatic function, and this could be promising for liver therapy. Lina R. Nih et al. used HA hydrogel that is both hyaluronidase biodegradable and matrix metalloproteinase (MMP) degradable as a scaffold to construct an artificial extracellular matrix to promote brain repair after stroke, and the scaffolds were loaded with VEGF loaded heparin nanoparticles (nH) [196]. The results showed that heparin granules could counteract the inflammatory effect caused by VEGF and lead to the formation of a pre-repair environment leading to the de novo formation of brain tissue [196]. This approach can directly produce neurovascular structure in the infarct cavity and modulate inflammatory, scar, and neural stem cell responses in the adjacent brain. Li, KY et al. seeded corneal endothelial cells (CEC) on fish scale collagen membrane (FSCM) and transplanted them [164]. After FSCM was implanted into the anterior chamber of rabbit cornea, it was found that the corneal transparency and thickness were normal at all time points, and no edema or turbidity was observed [164]. FSCM was suitable as a cell carrier for corneal transplantation. In conclusion, in a wide range of biomedical fields, the superior characteristics of marine materials need to be explored and utilized by the majority of scientific researchers to design the performance required in the corresponding field to fill the field of tissue engineering.

## 4. Conclusions and Prospects

The use of marine materials has undergone a long process. Marine biological materials have become a research hotspot in the field of materials science and biomedicine as they might have novel characteristics and unique biochemical properties. This attention is also attributed to the research and technological progress in material extraction and processing. There have been detailed studies on the activities of marine biological materials such as enhancing immunity, antitumor, lowering blood lipid, antioxidation, and anticoagulation [197,198,199]. However, due to the lack of industrial-scale extraction and purification of many of these compounds, there are few market-oriented products. Medical devices and advanced therapy medicinal products need to undergo very demanding regulation, so the realization of the clinical potential of marine materials will be a long and challenging process [200]. It is exciting that with the development of science and technology and the application of various new technologies, marine biological materials will be expected to achieve industrialization, development of nutraceuticals [199], drugs [201], and medical devices [202] and realize the high-value utilization of marine biology. 

While promoting the development, commercialization, and application of marine biomedical materials, their safety is also a concern. Marine biomedical materials mostly contain active biological macromolecules with unknown immunogenicity, and their composition and molecular structure will also affect their applicability and safety [1]. Due to strict biosafety requirements, the applications of these biomaterials in the biomedical field are not easy. Hence, we should establish physical and chemical characterization methods suitable for marine biomaterials and medical devices, according to the expected use of biomaterials, the contact mode with the human body, and the contact time, etc. It is of great significance to the development and use of marine materials. 

Continuing research on the oceans has revealed a wide range of biomedical materials with excellent properties, and we should continue to optimize the exploration of marine resources to ensure the sustainability of the identified biomedical material production [203]. Fully considering the actual clinical needs and developing novel materials based on the combination of biology and advanced methods, the research on marine biomedical materials will better benefit humankind.

## Figures and Tables

**Table 2:** Biomaterials from marine organisms applied in tissue engineering.

Materials	Marine Biomaterial Sources	Testing Cell Source or Active Ingredient	Forming Method	Application	Advantages (A) and Disadvantages (D)	Ref.
Alginate/gelation/ECM	Brown algae	Human HepaRG liver cells	Extrusion 3D printing	Liver tissue engineering	A: Improved cell viability and hepatic metabolic activity; high precision and stability of the printed constructs; D: Prevented cell–cell connection and influenced the measured metabolic activity of hepatocytes	[150]
Collagen	jellyfish *Rhopilema esculentum*	Human and rat chondrocytes	Freeze-drying	Cartilage tissue engineering	A: Safe, no cytotoxic effects, biocompatible, and a continuous biodegradability rate D: Persisting chronic inflammatory reactions within the vicinity of implants	[151]
Carrageenan/PVA	Red algae	Mesenchymal stem cells	Freeze-drying	Cartilage tissue engineering	A: Cell viability and the increase in proliferation; Imitated the structural features of natural cartilage	[152]
Alginate/gelatin	Brown algae	10T1/2 and HAE cells	Enzyme-catalyzed cross-linking	Fabrication of cell sheets and spherical tissues	A: Shorter time for enclosed cell growth; enhanced cell adhesion; maintaining on-demand degradability D: Reduced the degradability by alginate lyase treatment	[153]
CS/CHS/PDLLA	Shell	NGF	Layer-by-layer and Electro-Static-assembly technique	Neural tissue engineering	A: Good mechanical properties and degradation properties; good biocompatibility with Schwann cells	[154]
Alginate/gelation	Brown algae	hMSCs	micro-extrusion 3D printing	Bone tissue engineering	A: Provided uniform macropores and different compressive moduli D: Cell viability decreased with an increase in compressive modulus of the scaffolds	[155]
CHS/hydroxyapatite	Shell	MC3T3-E1	Extrusion 3D printing	Bone tissue engineering	A: Good mechanical support after printing and provided highly active cell-platforms D: Low mechanical strength and poor mechanical stabilities	[156]
SF/CS/HA	/	L929	Freeze-drying	Dermal tissue engineering	A: Contributed to blood capillary network formation; stimulated repair cells to secrete and enrich growth factors	[157]
Collagen/CHS	Blue shark (*Prionace glauca*)	6T-CEM and hFOB12	Freeze-drying	Bone tissue engineering	A: Compact, regular pore shapes; good biocompatibility and osteogenesis properties D: Fast degradation speed, reduced water binding capacity and shrinkage factor	[158]
Chitin	*I. basta* sponge skeletons	hBMSCs and human dermal MSCs	Decellularization and demineralization	Tissue engineering	A: Simplicity and ease of the isolation; interconnected porosity; excellent biocompatibility; D: Weakened cell attachment and viability after thawing	[159]
CHS/collagen	Shell/salmon skins	MSCs	Freeze-drying	Bone and cartilage tissue engineering	A: Enhanced the mechanical properties; enhances both MSC osteogenesis and chondrogenesis. D: Lower mechanical properties and decreased mean pore size	[160]
Collagen	Shark Skin	Chondrocyte cells (ATDC5)	Freeze-drying, Supercritical fluids	Cartilage tissue engineering	A: Highly porous and interconnected; Allows the cell adhesion, growth, and proliferation D: Low mechanical strength and fast degradation speed	[161]
Alginate/gelation	Brown algae	L929 and smooth muscle cells	3D printing	Vessel tissue engineering	A: Structures with multilevel fluidic channels; sufficient mechanical strength; exhibits biocompatibility D: Lower mechanical strength	[162]
Collagen/PLLA	Fish	Intestinal organoids	Solvent casting	Intestine tissue engineering	A: Beneficial in trapping the seeded cells, enhanced cell viability and growth, biofunctionality D: Weak mechanical nature and slower degradation speed	[163]
PCL/collagen	Fish-Scale	corneal endothelial cells	Cross-linked	Ocular tissue engineering	A: Suitable spherical curvature, transparent and biocompatible	[164]
Alginate	Brown algae	ZnO NPs	Ionic cross-linked	Dermal tissue engineering	A: Durable antibacterial; allows accessible mobility of molecular exchange required for improving chronic wound healing D: Slightly influenced cell viability	[165]
Alginate/Gelatin	Brown algae	human dental pulp stem cells (hDPSCs)	3D printing	Dental tissue engineering	A: Suitable for the growth of hDPSCs; promoted cell proliferation and differentiation	[166]
collagen	Salmon	HUVEC	Chemical cross-linked	Vessel tissue engineering	A: Biodegradability; enhanced the production of inflammatory cytokines in HUVECs	[167]
HS	Mollusk *Nodipecten nodosus*	/	Enzymatic Treatments	Anticoagulant drug	A: Inhibited thrombus growth in photochemically injured arteries D: Limited sources and toxicity	[168]
Collagen/PLGA	Tilapia skin	/	Self-assembly; electrospinning	Tissue engineering	A: Good biocompatibility and immunogenicity; good hemostatic function; guided bone regeneration D: Low mechanical strength and induced some immunogenicity	[169]

## Abbreviations

Hydrogen peroxide(H_2_O_2_); ethylenediamine tetraacetic acid (EDTA); degree of acetylation (DA); molecular weight (MW); chitosan (CHS); β-d-mannuronic acid (M unit); α-l-guluouronic acid (G unit); microwave assisted extraction (MAE); ultrasonic assisted extraction (UAE); pressurized liquid extraction (PLE); enzyme extraction (EAE); glycosaminoglycan (GAG); deep eutectic solvents (DES); ulvanobiuronic acid 3-sulfate (A_3s_); ulvanobiuronic acid 3-sulfate (B_3s_); carrageenan (CG); hyaluronic acid (HA); chondroitin sulfate (CS); heparin/heparin sulfate (HS); dermatan sulfate (DS); keratan sulfate (KS); d-glucuronic (GlcA); N-acety1-galactosamine (GalNAc); l-iduronic acid (IdoA); d-glucosamine (GlcN); d-galactose (Gal); fraction V (FV); homogenization-assisted extraction (HAE); silk fibroin (SF); 5—fluorouracil (5—Fu); poly(ε-caprolactone) (PCL); three dimensions (3D); nanoparticles (NPs); mesenchymal stem cells (MSCs); extracellular matrix (ECM); synovial MSCs (SMSCs); senescence marker protein (SMP-30); recombinant human bone morphogenetic protein-2 (rhBMP-2); nerve growth factor (NGF); vascular endothelial growth factor (VEGF); tetrahedral framework nucleic acid (TFNA); matrix metalloproteinase (MMP); heparin nanoparticles (nH); corneal endothelial cells (CEC); fish scale collagen membrane (FSCM).
